# Supplementary material for: Decoding critical long non-coding RNA in ovarian cancer epithelial-to-mesenchymal transition
Source: Nat Commun. 2017 Nov 17;8:1604. doi: 10.1038/s41467-017-01781-0 (PMC5693921; doi:10.1038/s41467-017-01781-0)
Supplement: Supplementary file 1 — Supplementary Information [file 41467_2017_1781_MOESM1_ESM.pdf]

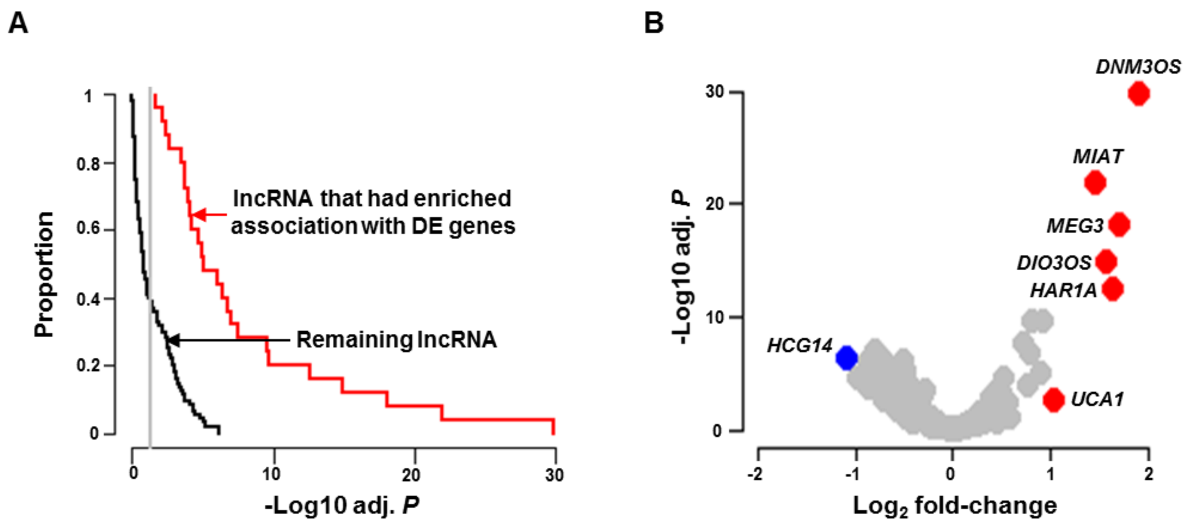

Supplementary Figure 1. LncRNA differentially expressed in EMT. A) Distribution of differential expression *P* values of 25 lncRNA inferred from the linear regression model (red line) and the remaining lncRNA (black line). X-axis represents significance level of differential expression. Y-axis represents proportion of lncRNA that are significantly differentially expressed. Grey vertical line represents adjusted *P* value threshold of 0.05 (i.e. 1.301 in $-\log_{10}$ scale). B) Seven lncRNA had at least 2 fold expression changes with adjusted $P<0.05$ in the mesenchymal subtype compared to the epithelial subtype. Red and blue dots represent significantly up- and down-regulated lncRNA, respectively. Remaining lncRNA are represented by grey dots. For A and B, *P* values determined by BH adjusted two-tailed t-test.

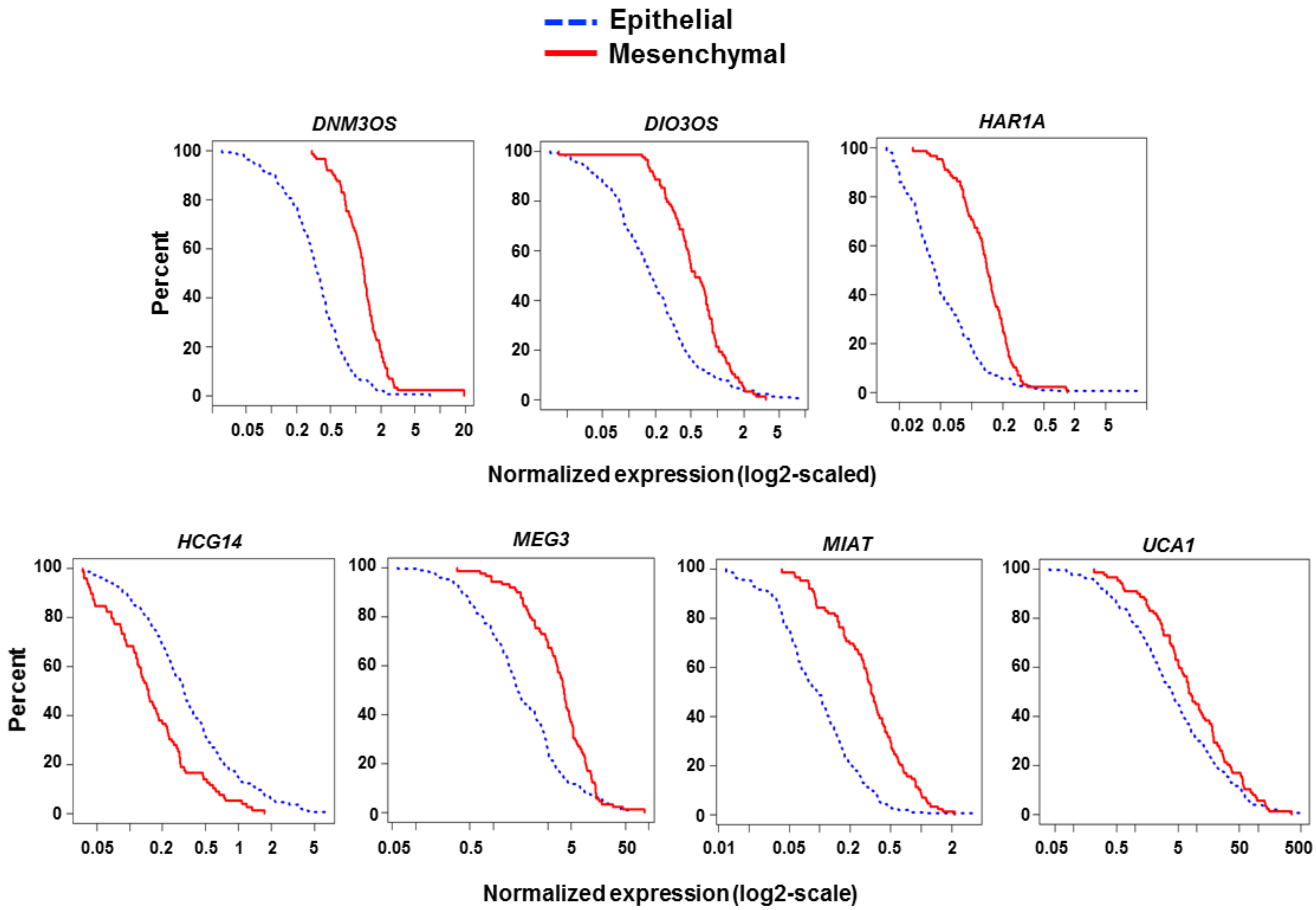

Supplementary Figure 2. Empirical cumulative distribution of lncRNA expression in epithelial and mesenchymal subtypes in TCGA cohort. Distribution of seven lncRNA expression in two ovarian cancer subtypes. X-axis represents normalized expression of indicated lncRNA. Y-axis estimates percentage of patient sample at or below a given value of lncRNA expression.

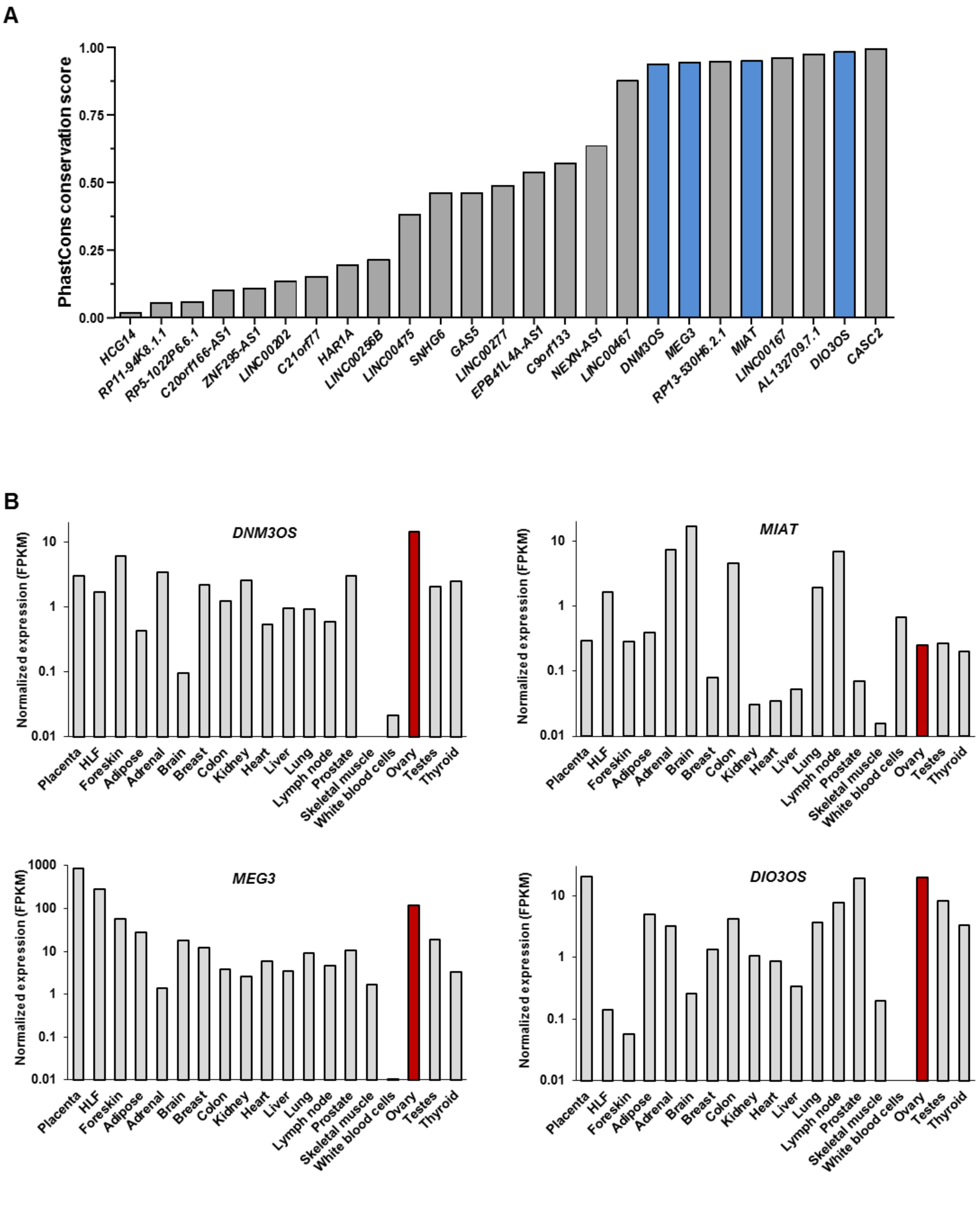

Supplementary Figure 3. Analysis of conservation and expression of selected lncRNA. A) Distribution of maximal conservation levels of lncRNA within 200 bp window. Blue bars denote those lncRNA that were strongly conserved across the primate species and potentially active in ovarian cancer EMT. B) Distribution of four lncRNA expression in 19 human normal tissues, including ovary.

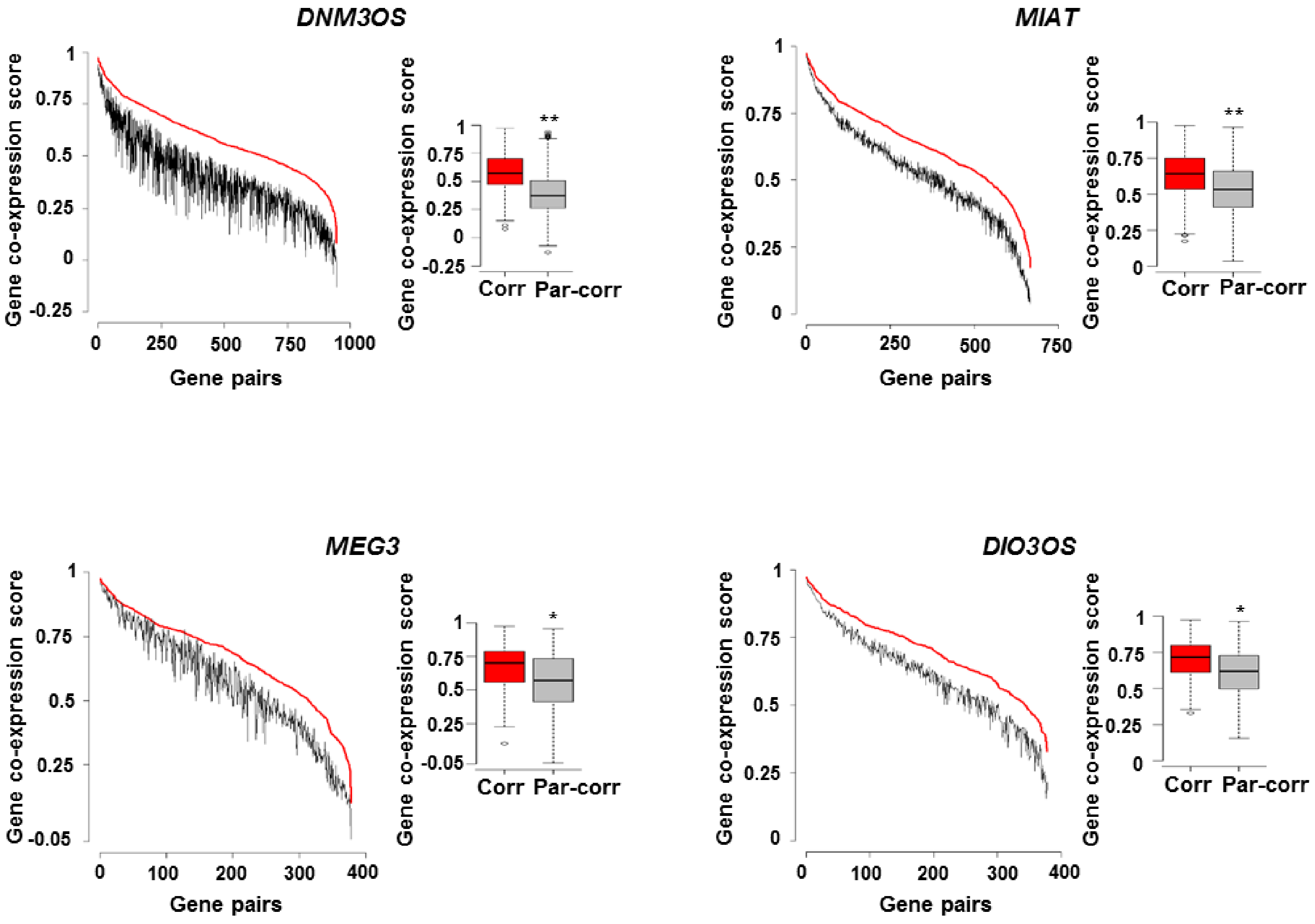

Supplementary Figure 4. LncRNA-dependent gene co-expression. Reduction of co-expression of EMT-linked genes after removing the effect of the indicated lncRNA using first-order partial correlation statistic. The analyses show comparative results of correlation and first-order partial correlation (Par-corr). Box and whisker plot summarizes the reduction of co-expression of EMT-linked genes after removing the effect of the lncRNA compared with the correlation value. Box represents the 25th and 75th percentiles, line indicates the median, and whiskers represent the maximum and minimum; $^{**}P<2.2\text{X}10^{-16}$; $^{*}P<2.1\text{X}10^{-11}$, Wilcoxon rank-sum test.

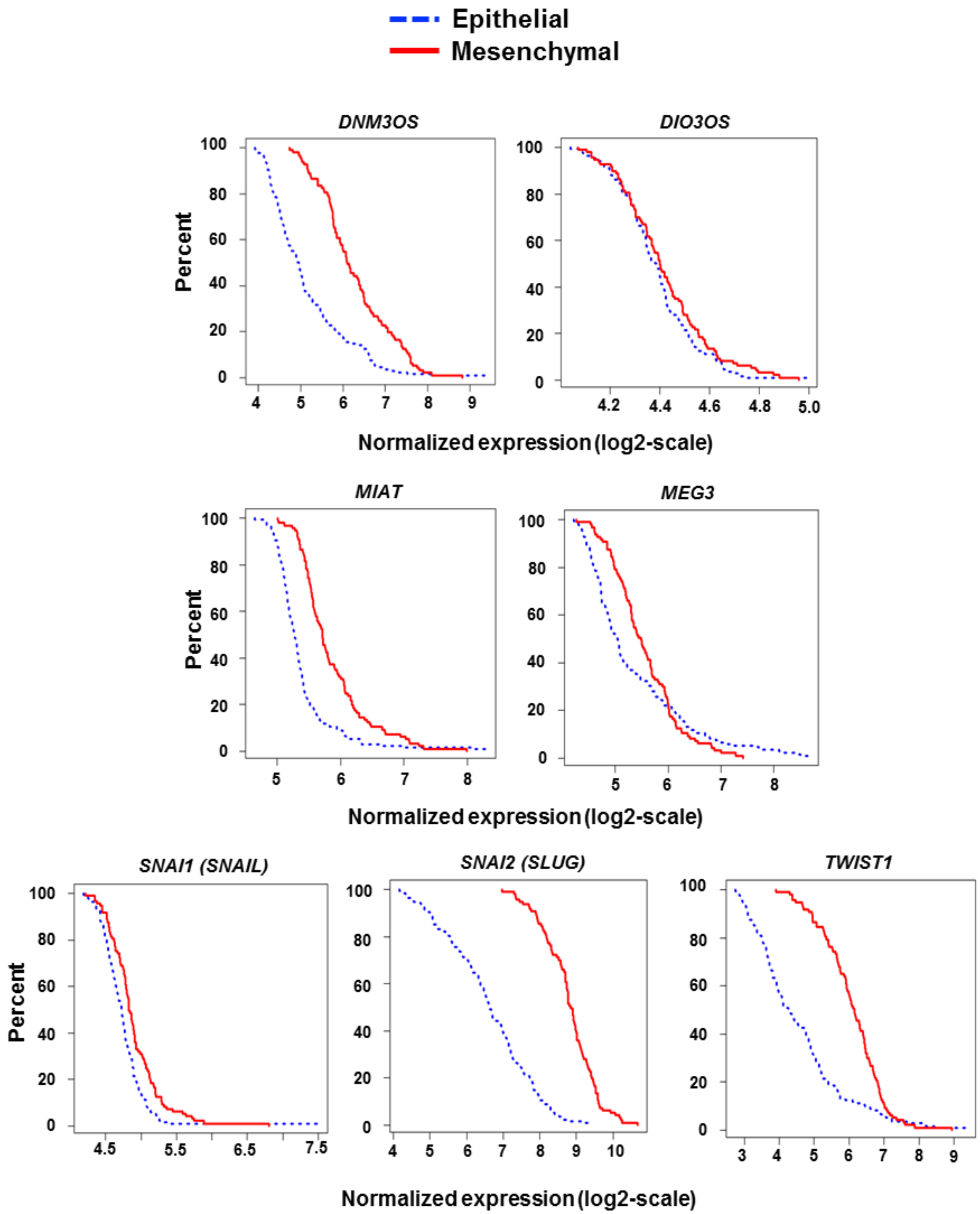

Supplementary Figure 5. Empirical cumulative distribution of lncRNA expression in epithelial and mesenchymal subtypes in GSE9891 patient cohort. Distribution of expression of four lncRNA and three EMT-linked mRNA in epithelial and mesenchymal ovarian cancer subtypes. X-axis represents normalized expression of indicated lncRNA or mRNA. Y-axis estimates percentage of patient sample at or below a given value of lncRNA or mRNA expression.

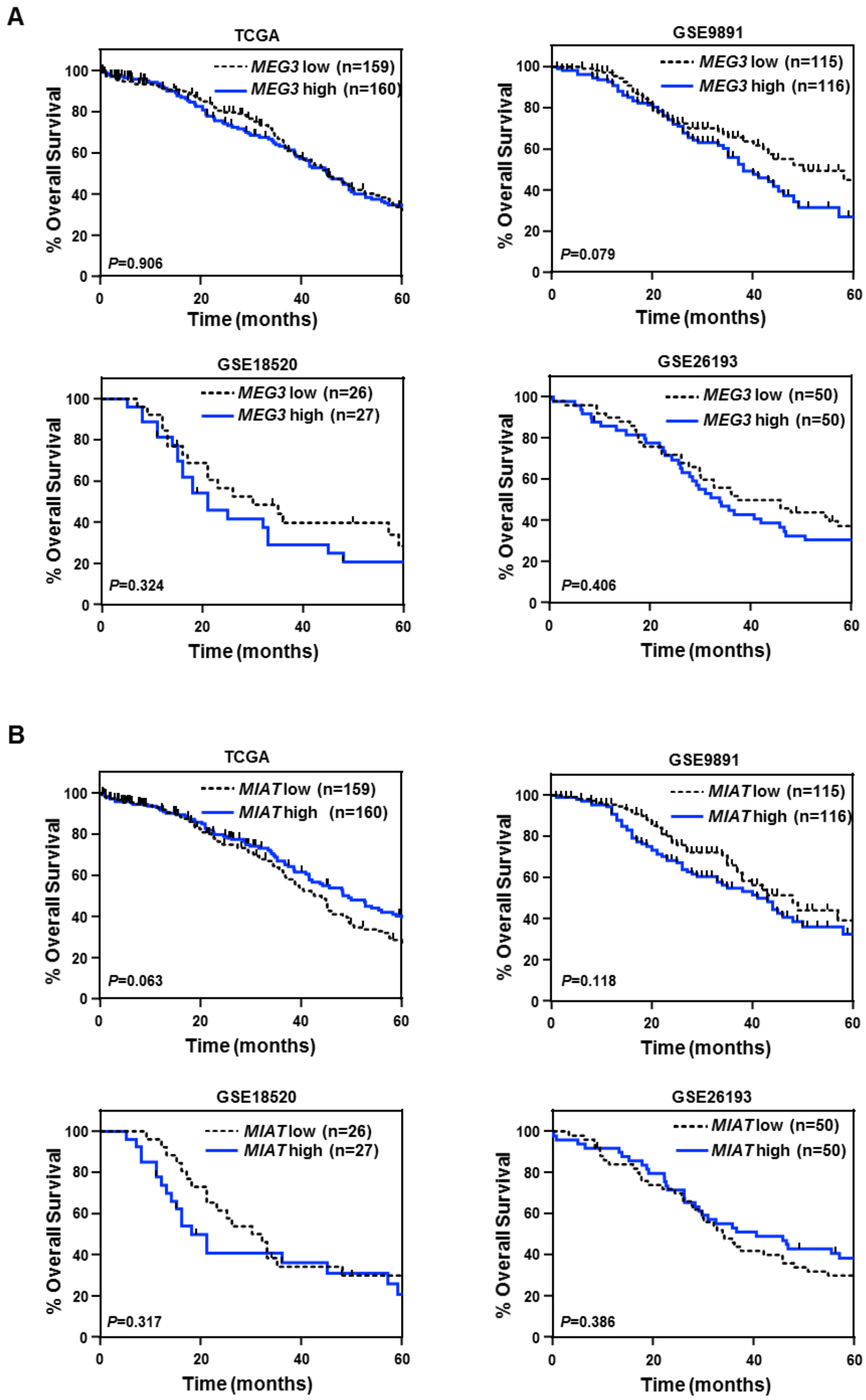

Supplementary Figure 6. No correlation of *MEG3* or *MIAT* expression and overall survival of ovarian cancer patients. Ovarian cancer patient samples were separated into A) *MEG3* low and *MEG3* high or B) *MIAT* low and *MIAT* high based on the median expression in four independent patient cohorts and Kaplan-Meier survival analyses were performed. *P*-values determined by log-rank test.

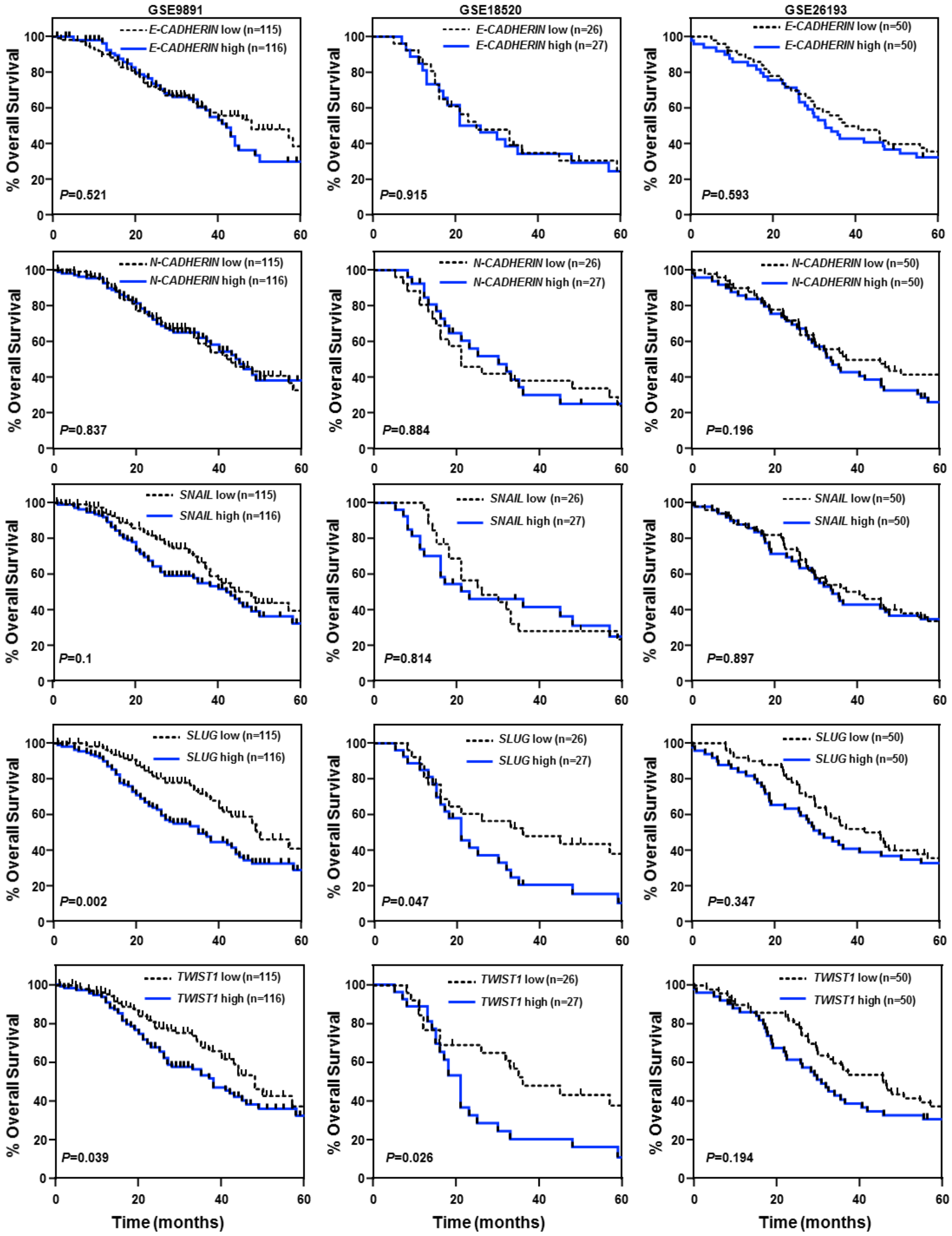

Supplementary Figure 7. Ovarian cancer patient overall survival and correlations with EMT genes. Ovarian cancer patient samples were separated into low and high levels of the indicated mRNA based on median expression in three independent patient cohorts and Kaplan-Meier survival analyses were performed. *P*-values determined by log-rank test.

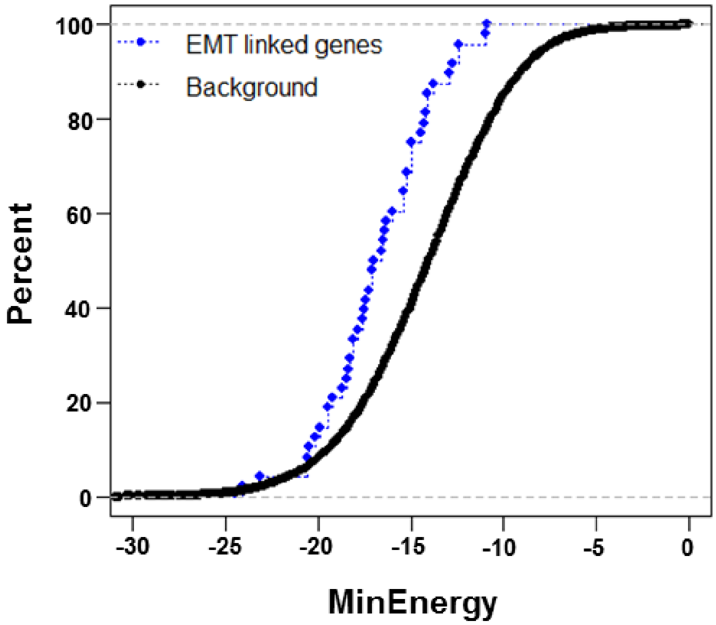

Supplementary Figure 8. Predicted binding affinity between *DNM3OS* and EMT linked genes. Empirical cumulative distribution of predicted interaction energy scores (MinEnergy) for *DNM3OS* with 48 EMT linked genes (blue dots) or background (genome-wide) gene set. X-axis represents minimum interaction energy (MinEnergy). Y-axis estimates percentage of genes for each group at or below a given MinEnergy score.

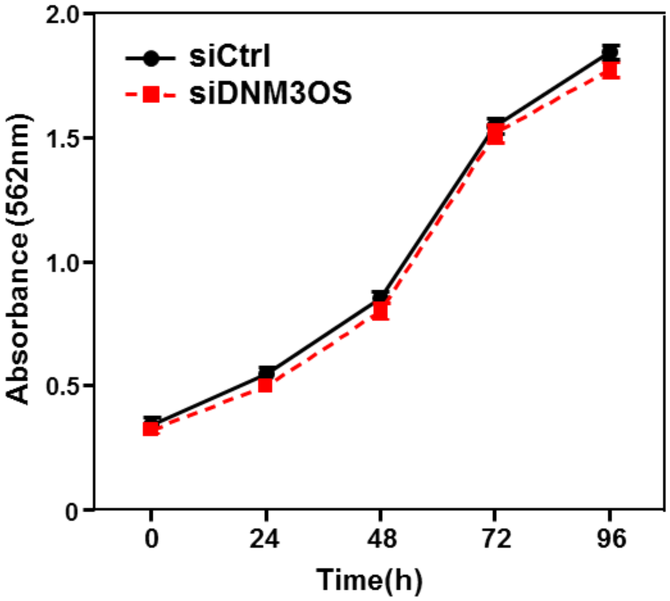

Supplementary Figure 9. ***DNM3OS*** **does not influence ovarian cancer proliferation.** MTT assays were performed at intervals starting 24 hrs after transfection of SKOV3 cells with SMARTpool siRNA or non-targeting control (triplicates); ±SEM. Data are representative of at least three independent experiments.

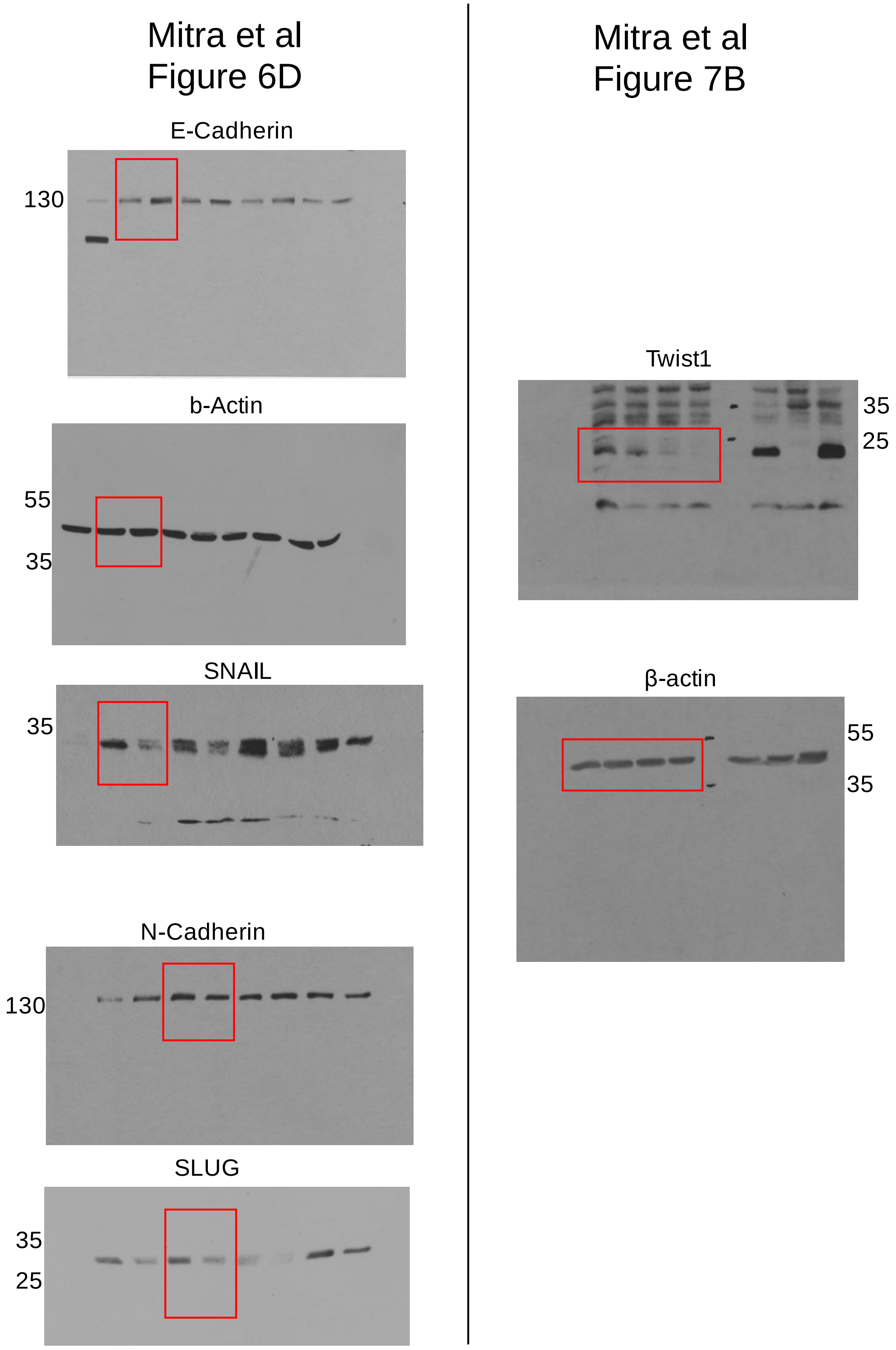

Supplementary Figure 10. Uncropped scans of the Western blots in the manuscript. Uncropped scans of the Western blots in Fig 6D and Fig 7B.

Supplementary Table 1. Use of TCGA high-grade serous ovarian cancer data

Data	Used for
lncRNA expression (RPKM[ǂ])	Multivariate regression<br>Differential expression<br>Expression correlation
mRNA expression (RPKM)	Multivariate regression<br>Expression correlation
mRNA expression (Read counts)	Differential expression
Copy number (Normalized segmented values)	Multivariate regression
Methylation (Beta-values)	Multivariate regression
miRNA expression (RPM[§])	Expression correlation
miRNA expression (Read counts)	Differential expression
Protein expression (iTRAQ[±])	Differential expression

[ǂ]RPKM – Reads per kilobase of transcript per million mapped reads; [§]RPM – Reads per million mapped reads; [±] isobaric tags for relative and absolute quantitation.

Supplementary Table 2. Differential expression of known EMT-linked genes[§]

Count	Gene symbol	Fold-change (log2)	*P*	Adj. *P*[*]
1	*POSTN*	3.671	1.57e-69	2.65e-67
2	*COL3A1*	3.297	6.19e-165	2.79e-161
3	*COL5A2*	3.228	2.22e-187	3.00e-183
4	*FN1*	3.218	9.61e-150	1.30e-146
5	*SERPINE1*	3.177	8.67e-112	5.10e-109
6	*COL8A1*	3.166	2.01e-110	1.09e-107
7	*VCAN*	3.157	4.47e-143	5.49e-140
8	*MMP2*	2.754	7.98e-150	1.20e-146
9	*LOX*	2.712	5.73e-108	2.77e-105
10	*COL1A2*	2.663	3.99e-130	3.37e-127
11	*SNAI2*	2.391	4.24e-101	1.68e-98
12	*SPARC*	2.383	6.16e-160	2.08e-156
13	*CXCL12*	2.342	7.51e-61	9.77e-59
14	*PDGFRB*	2.004	2.82e-97	1.03e-94
15	*MMP14*	1.883	1.14e-85	3.02e-83
16	*LOXL2*	1.881	7.85e-103	3.22e-100
17	*PDPN*	1.876	1.12e-44	1.02e-42
18	*ITGA5*	1.780	1.90e-96	6.75e-94
19	*CTGF*	1.777	1.08e-41	9.04e-40
20	*CYR61*	1.767	3.39e-45	3.16e-43
21	*TNC*	1.705	1.02e-24	4.91e-23
22	*TGFB1I1*	1.595	3.14e-72	5.74e-70
23	*CDH13*	1.547	1.94e-21	8.20e-20
24	*THBD*	1.547	2.54e-40	2.03e-38
25	*ZEB1*	1.485	7.81e-57	9.27e-55
26	*TGFB3*	1.469	1.20e-28	6.56e-27
27	*ZEB2*	1.449	1.22e-51	1.29e-49
28	*TWIST1*	1.440	3.28e-18	1.24e-16
29	*BMP4*	1.411	1.77e-18	6.76e-17
30	*CALD1*	1.359	4.28e-60	5.41e-58
31	*IGFBP4*	1.314	1.18e-39	9.33e-38
32	*SDC1*	1.280	4.17e-19	1.62e-17
33	*RGS2*	1.269	4.06e-20	1.66e-18
34	*SNAI1*	1.218	1.07e-23	4.98e-22
35	*DDR2*	1.202	1.03e-16	3.46e-15
36	*SEMA7A*	1.199	2.64e-34	1.81e-32
37	*DAB2*	1.194	8.36e-41	6.77e-39
38	*TFPI2*	1.173	3.11e-08	3.67e-07
39	*COL8A2*	1.163	2.03e-16	6.66e-15
40	*PTGS2*	1.056	1.10e-10	2.05e-09
41	*MMP9*	1.032	2.65e-07	2.47e-06
42	*PLAUR*	1.009	7.20e-24	3.41e-22
43	*BMP1*	1.001	5.69e-32	3.56e-30
44	*TP73*	-1.052	2.41e-08	2.92e-07

[§]Literature supported EMT-linked genes were extracted from Chanrion et. al. [1] and Zhao et. al. [2];
[*]*P*-values were adjusted using the Benjamini-Hochberg [3] multiple testing correction method.

References for Supplementary Table 2.

1. Chanrion M et. al. Concomitant Notch activation and p53 deletion trigger epithelial-to-mesenchymal transition and metastasis in mouse gut. Nature communications. 2014; 5:5005.
2. Zhao M, Kong L, Liu Y and Qu H. dbEMT: an epithelial-mesenchymal transition associated gene resource. Scientific reports. 2015; 5:11459.
3. Benjamini Y and Hochberg Y. Controlling the False Discovery Rate - a Practical and Powerful Approach to Multiple Testing. J Roy Stat Soc B Met. 1995; 57(1):289-300.

Supplementary Table 3. Linear expression association with survival time

Data set	lncRNA	Spearman's ρ	Correlation *P*
GSE9891	*DNM3OS*	-0.110	0.048
	MEG3	0.0004	0.502
	MIAT	-0.040	0.273
GSE18520	*DNM3OS*	-0.252	0.034
	MEG3	-0.061	0.332
	MIAT	-0.241	0.041
GSE26193	*DNM3OS*	-0.281	0.002
	MEG3	-0.129	0.101
	MIAT	-0.074	0.230

Supplementary Table 4. Differentially expressed genes in TCGA cohort that are significantly associated with *DNM3OS*

Count	Gene symbol	*P*-value	BH adjusted *P*-value
1	*ACTA2*	1.84E-26	1.76E-23
2	*ACTG2*	2.36E-27	2.60E-24
3	*ADAM12*	2.76E-31	5.24E-28
4	*ADAM19*	8.77E-21	3.62E-18
5	*ADAMTS10*	4.60E-38	2.27E-34
6	*ADAMTS14*	2.33E-18	6.56E-16
7	*ADAMTS2*	3.66E-32	7.77E-29
8	*ADAMTS4*	7.16E-21	3.00E-18
9	*ADAMTS5*	5.21E-17	1.15E-14
10	*ADAMTSL1*	1.17E-20	4.75E-18
11	*ADAMTSL3*	1.75E-13	1.92E-11
12	*ADRA2A*	5.60E-16	1.00E-13
13	*AEBP1*	2.47E-44	2.18E-40
14	*ALDH1A3*	2.87E-16	5.44E-14
15	*ANGPTL2*	6.52E-41	4.13E-37
16	*ANTXR1*	1.12E-44	1.02E-40
17	*AOC3*	2.86E-13	3.01E-11
18	*APLNR*	2.54E-22	1.34E-19
19	*APOD*	4.85E-21	2.10E-18
20	*ATP10A*	1.06E-11	7.89E-10
21	*BASP1*	2.69E-15	4.22E-13
22	*BMP4*	1.76E-15	2.86E-13
23	*C10orf55*	1.68E-11	1.19E-09
24	*C13orf33*	1.48E-17	3.60E-15
25	*C1QTNF1*	3.83E-41	2.52E-37
26	*C1QTNF3*	1.03E-18	3.06E-16
27	*C1QTNF6*	1.09E-26	1.08E-23
28	*C7orf10*	6.92E-10	3.30E-08
29	*CACNA1C*	4.24E-33	9.89E-30
30	*CALD1*	1.29E-33	3.23E-30
31	*CCDC80*	3.64E-22	1.87E-19
32	*CD163*	5.95E-12	4.68E-10
33	*CD209*	5.20E-12	4.14E-10
34	*CD248*	2.37E-24	1.65E-21
35	*CD36*	1.73E-12	1.53E-10
36	*CD93*	4.42E-25	3.42E-22
37	*CDH11*	1.04E-54	2.44E-50
38	*CERCAM*	4.19E-18	1.13E-15
39	*CHRD*	6.58E-44	5.54E-40
40	*CILP*	1.30E-16	2.63E-14
41	*CILP2*	7.99E-20	2.83E-17
42	*CLDN11*	4.63E-29	6.60E-26

43	*CNN1*	3.00E-23	1.78E-20
44	*CNN2*	1.31E-21	6.18E-19
45	*CNRIP1*	1.01E-71	9.07E-67
46	*COL10A1*	7.34E-25	5.52E-22
47	*COL11A1*	6.70E-24	4.40E-21
48	*COL15A1*	8.06E-32	1.63E-28
49	*COL1A1*	3.63E-51	5.59E-47
50	*COL1A2*	5.31E-55	1.43E-50
51	*COL3A1*	7.35E-49	9.43E-45
52	*COL4A1*	6.19E-22	3.09E-19
53	*COL5A1*	4.39E-51	6.57E-47
54	*COL5A2*	7.75E-44	6.43E-40
55	*COL5A3*	5.68E-20	2.07E-17
56	*COL6A1*	2.88E-45	2.82E-41
57	*COL6A2*	2.08E-51	3.40E-47
58	*COL6A3*	2.84E-61	1.39E-56
59	*COL8A1*	2.10E-33	5.08E-30
60	*COL8A2*	1.48E-13	1.65E-11
61	*COLEC12*	8.82E-35	2.58E-31
62	*COMP*	1.13E-11	8.35E-10
63	*CSDC2*	1.20E-12	1.10E-10
64	*CSF2RB*	4.54E-12	3.66E-10
65	*CTGF*	1.62E-23	1.00E-20
66	*CTHRC1*	4.91E-26	4.44E-23
67	*CTSK*	1.23E-28	1.64E-25
68	*CXCL12*	3.70E-24	2.52E-21
69	*CXCL14*	3.64E-17	8.27E-15
70	*CYP1B1*	5.99E-11	3.73E-09
71	*CYR61*	4.44E-22	2.26E-19
72	*DAB2*	1.99E-30	3.45E-27
73	*DACT1*	3.18E-46	3.43E-42
74	*DACT3*	2.30E-40	1.41E-36
75	*DCHS1*	9.79E-35	2.85E-31
76	*DCLK1*	1.13E-14	1.57E-12
77	*DCN*	1.79E-52	3.45E-48
78	*DDR2*	9.42E-23	5.23E-20
79	*DES*	1.21E-11	8.89E-10
80	*DKK2*	2.97E-28	3.78E-25
81	*DPYSL3*	1.16E-16	2.37E-14
82	*ECM1*	1.13E-28	1.52E-25
83	*EFEMP1*	3.59E-11	2.36E-09
84	*EGR2*	6.08E-12	4.77E-10
85	*EHD2*	2.12E-18	5.98E-16
86	*ELN*	2.54E-16	4.87E-14
87	*EMILIN1*	3.45E-59	1.33E-54

88	*ENPP1*	3.13E-13	3.26E-11
89	*ETV1*	3.03E-34	8.25E-31
90	*F13A1*	5.62E-28	6.87E-25
91	*F2R*	9.76E-25	7.17E-22
92	*FAM101A*	8.06E-19	2.44E-16
93	*FAM198B*	6.46E-55	1.66E-50
94	*FAP*	1.31E-27	1.52E-24
95	*FBLN2*	9.14E-12	6.89E-10
96	*FBN1*	4.19E-58	1.33E-53
97	*FEZ1*	3.37E-27	3.64E-24
98	*FIBIN*	2.23E-29	3.33E-26
99	*FILIP1L*	2.02E-51	3.40E-47
100	*FKBP14*	2.36E-32	5.09E-29
101	*FLNC*	3.53E-13	3.63E-11
102	*FLRT2*	6.97E-32	1.42E-28
103	*FN1*	4.26E-27	4.48E-24
104	*FOLR2*	3.78E-13	3.86E-11
105	*FPR1*	1.23E-11	9.01E-10
106	*FRMD6*	3.91E-39	2.13E-35
107	*FSTL1*	1.33E-34	3.83E-31
108	*FSTL3*	8.34E-20	2.94E-17
109	*FZD1*	4.20E-29	6.07E-26
110	*GAS1*	8.20E-10	3.84E-08
111	*GAS7*	7.19E-24	4.70E-21
112	*GEM*	2.11E-19	7.00E-17
113	*GFPT2*	9.56E-25	7.04E-22
114	*GHR*	3.29E-12	2.74E-10
115	*GJB2*	1.56E-14	2.12E-12
116	*GLI3*	6.69E-22	3.32E-19
117	*GLT8D2*	4.78E-48	5.73E-44
118	*GPR124*	7.54E-52	1.35E-47
119	*GUCY1A3*	1.23E-14	1.70E-12
120	*HIC1*	1.55E-56	4.64E-52
121	*HOPX*	9.15E-22	4.44E-19
122	*HTRA1*	1.98E-34	5.56E-31
123	*HTRA3*	3.00E-33	7.16E-30
124	*ID3*	3.53E-17	8.04E-15
125	*IGFBP4*	8.97E-41	5.62E-37
126	*IL16*	1.79E-14	2.40E-12
127	*INHBA*	1.53E-26	1.48E-23
128	*ISLR*	6.39E-41	4.10E-37
129	*ITGA11*	2.48E-43	2.00E-39
130	*ITGA5*	1.70E-31	3.31E-28
131	*JAM3*	4.22E-34	1.12E-30
132	*JPH2*	3.93E-15	5.97E-13

133	*KCNE4*	1.58E-42	1.22E-38
134	*KCNMB1*	1.64E-14	2.22E-12
135	*KIFC3*	1.16E-21	5.52E-19
136	*KLF2*	1.86E-19	6.22E-17
137	*LAMA4*	6.94E-31	1.26E-27
138	*LAMB1*	8.54E-28	1.02E-24
139	*LDB2*	3.85E-30	6.43E-27
140	*LHFP*	9.73E-59	3.28E-54
141	*LMOD1*	8.86E-36	3.14E-32
142	*LOX*	4.77E-29	6.78E-26
143	*LOXL1*	2.75E-27	2.99E-24
144	*LOXL2*	3.04E-37	1.33E-33
145	*LRP1*	1.66E-21	7.70E-19
146	*LRRC17*	1.70E-20	6.73E-18
147	*LRRN4CL*	3.04E-40	1.84E-36
148	*LTBP2*	4.08E-21	1.79E-18
149	*LUM*	1.25E-33	3.15E-30
150	*LYVE1*	2.18E-11	1.51E-09
151	*LZTS1*	1.85E-25	1.53E-22
152	*MATN3*	4.22E-26	3.87E-23
153	*MDFI*	9.47E-20	3.32E-17
154	*MEIS3*	3.12E-39	1.73E-35
155	*MFAP5*	7.41E-11	4.52E-09
156	*MME*	1.56E-19	5.28E-17
157	*MMP11*	9.55E-26	8.26E-23
158	*MMP14*	3.04E-31	5.75E-28
159	*MMP2*	4.89E-40	2.83E-36
160	*MN1*	3.73E-23	2.18E-20
161	*MOXD1*	5.00E-17	1.11E-14
162	*MRC2*	8.50E-24	5.53E-21
163	*MRGPRF*	3.20E-48	3.92E-44
164	*MRVI1*	1.73E-55	4.91E-51
165	*MSC*	1.03E-28	1.38E-25
166	*MSRB3*	2.19E-41	1.46E-37
167	*MYH11*	2.63E-17	6.14E-15
168	*NAV3*	2.02E-37	9.23E-34
169	*NBL1*	4.01E-22	2.05E-19
170	*NEDD4*	1.93E-17	4.59E-15
171	*NID2*	3.26E-34	8.83E-31
172	*NKD2*	1.64E-27	1.86E-24
173	*NT5E*	1.08E-22	5.98E-20
174	*NUAK1*	3.06E-31	5.77E-28
175	*OLFML1*	1.61E-51	2.80E-47
176	*OLFML2B*	1.88E-27	2.10E-24
177	*OLFML3*	2.58E-23	1.54E-20

178	*PALLD*	1.52E-30	2.69E-27
179	*PAPSS2*	7.83E-29	1.08E-25
180	*PCOLCE*	7.30E-36	2.66E-32
181	*PDGFD*	4.27E-14	5.33E-12
182	*PDGFRA*	4.80E-60	1.99E-55
183	*PDGFRB*	5.36E-49	7.05E-45
184	*PDLIM3*	8.01E-35	2.41E-31
185	*PDPN*	3.15E-30	5.32E-27
186	*PHLDB2*	2.05E-14	2.72E-12
187	*PLAU*	3.28E-17	7.52E-15
188	*PLK2*	1.17E-16	2.39E-14
189	*PLXDC1*	2.84E-36	1.13E-32
190	*PMEPA1*	2.71E-17	6.31E-15
191	*PMP22*	2.31E-27	2.56E-24
192	*PODN*	8.58E-34	2.19E-30
193	*PODNL1*	9.06E-13	8.54E-11
194	*POSTN*	2.03E-17	4.80E-15
195	*PRDM1*	3.73E-24	2.53E-21
196	*PRRX1*	6.86E-35	2.15E-31
197	*PTGER3*	5.91E-14	7.17E-12
198	*PTGIS*	6.92E-36	2.54E-32
199	*PTRF*	2.17E-19	7.19E-17
200	*PXDN*	2.24E-15	3.58E-13
201	*RAB31*	4.45E-29	6.40E-26
202	*RASGRF2*	3.95E-50	5.75E-46
203	*RCAN2*	5.20E-25	3.99E-22
204	*RCN3*	1.33E-27	1.54E-24
205	*RGS16*	2.77E-16	5.27E-14
206	*RGS2*	3.46E-11	2.28E-09
207	*RGS4*	1.06E-42	8.28E-39
208	*RUNX2*	2.35E-24	1.64E-21
209	*SCARF2*	2.44E-29	3.62E-26
210	*SCUBE2*	1.06E-13	1.22E-11
211	*SDC1*	1.90E-13	2.07E-11
212	*SEMA3C*	2.80E-21	1.27E-18
213	*SEMA7A*	1.89E-32	4.09E-29
214	*SERPINE1*	9.95E-27	9.95E-24
215	*SERPINF1*	7.62E-37	3.26E-33
216	*SFRP1*	3.90E-12	3.19E-10
217	*SFRP2*	1.62E-15	2.65E-13
218	*SFRP4*	1.00E-49	1.42E-45
219	*SLC2A3*	2.99E-13	3.13E-11
220	*SLIT2*	1.36E-11	9.85E-10
221	*SMOC2*	1.82E-34	5.14E-31
222	*SNAI2*	8.72E-59	3.13E-54

223	*SPARC*	1.07E-44	9.94E-41
224	*SPHK1*	5.67E-17	1.24E-14
225	*SPOCK1*	1.63E-11	1.16E-09
226	*SPON2*	6.13E-27	6.29E-24
227	*ST6GAL2*	7.20E-10	3.42E-08
228	*SULF1*	8.67E-11	5.19E-09
229	*SULF2*	5.92E-36	2.20E-32
230	*SYNE1*	1.46E-22	7.93E-20
231	*TAGLN*	1.31E-25	1.11E-22
232	*TDO2*	8.80E-13	8.33E-11
233	*TGFB1I1*	5.25E-25	4.03E-22
234	*TGFB3*	9.68E-26	8.36E-23
235	*TGFBI*	6.45E-29	9.01E-26
236	*THBD*	1.74E-16	3.45E-14
237	*THBS1*	1.03E-37	4.91E-34
238	*THBS2*	1.85E-36	7.50E-33
239	*TIMP2*	1.01E-32	2.25E-29
240	*TIMP3*	1.39E-20	5.57E-18
241	*TMEM119*	3.51E-19	1.13E-16
242	*TMEM158*	3.39E-12	2.81E-10
243	*TMEM200A*	2.52E-42	1.89E-38
244	*TNC*	2.81E-14	3.63E-12
245	*TNFAIP6*	8.83E-14	1.03E-11
246	*TNFSF4*	1.34E-19	4.57E-17
247	*TSHZ3*	3.79E-29	5.51E-26
248	*TSPAN18*	5.35E-18	1.40E-15
249	*TWIST1*	9.01E-23	5.03E-20
250	*VCAM1*	1.21E-25	1.03E-22
251	*VCAN*	2.34E-38	1.18E-34
252	*WISP1*	1.10E-46	1.24E-42
253	*XYLT1*	9.19E-18	2.31E-15
254	*ZCCHC24*	1.06E-35	3.66E-32
255	*ZEB1*	8.88E-62	5.32E-57
256	*ZEB2*	1.72E-31	3.34E-28

Supplementary Table 5. Protein-protein interactions between *DNM3OS*-associated protein coding genes

Count	Interactor A	Interactor B
1	ACTA2	CNN2
2	ADAM12	FSTL3
3	ADAMTS4	FN1
4	BMP4	FSTL1
5	BMP4	HTRA1
6	CD36	CHRD
7	COL1A1	CD36
8	COL1A1	CD93
9	COL1A1	COL1A2
10	COL1A1	CTHRC1
11	COL1A1	DCN
12	COL1A1	DDR2
13	COL1A1	FN1
14	COL1A1	HTRA1
15	COL1A1	ITGA5
16	COL1A1	LOX
17	COL1A1	MMP2
18	COL1A1	NID2
19	COL1A1	SPARC
20	COL1A1	TGFBI
21	COL1A1	THBS1
22	COL1A2	CD36
23	COL1A2	CD93
24	COL1A2	COL5A3
25	COL1A2	LOX
26	COL1A2	LUM
27	COL1A2	SPARC
28	COL1A2	TGFBI
29	COL3A1	DDR2
30	COL3A1	HTRA1
31	COL3A1	LOX
32	COL3A1	PCOLCE
33	COL3A1	SPARC
34	COL3A1	THBS1
35	COL4A1	CD93
36	COL4A1	DCN
37	COL4A1	FBLN2
38	COL4A1	FN1
39	COL4A1	NID2
40	COL4A1	TGFBI

41	COL4A1	THBS1
42	COL5A1	COL5A3
43	COL8A2	COL8A1
44	CTGF	LRP1
45	DAB2	LRP1
46	DCN	COL1A2
47	DCN	COL5A1
48	DCN	COL6A1
49	DCN	ELN
50	DCN	FBN1
51	DCN	MMP2
52	DCN	THBS1
53	ELN	FBLN2
54	ELN	FBN1
55	ELN	LOX
56	ELN	LOXL1
57	ELN	NID2
58	F13A1	FN1
59	F2R	CHRD
60	FBN1	FBLN2
61	FBN1	MFAP5
62	FEZ1	OLFML3
63	FLNC	PHLDB2
64	FN1	CNN2
65	FN1	COL1A2
66	FN1	COL6A2
67	FN1	COMP
68	FN1	CTGF
69	FN1	CXCL12
70	FN1	DAB2
71	FN1	DCN
72	FN1	FBLN2
73	FN1	FLNC
74	FN1	ITGA5
75	FN1	LOX
76	FN1	NT5E
77	FN1	SPARC
78	FN1	TGFBI
79	FN1	THBS1
80	FN1	TNC
81	FSTL1	INHBA
82	FSTL3	FN1
83	FSTL3	INHBA

84	GLI3	TWIST1
85	ITGA5	CTGF
86	ITGA5	TNC
87	LAMB1	COL6A1
88	LOX	FBN1
89	MATN3	COMP
90	MME	MRVI1
91	MMP14	LRP1
92	MMP14	LUM
93	MMP14	SPOCK1
94	MMP2	COL6A3
95	MMP2	CXCL12
96	MMP2	MMP14
97	MMP2	SPOCK1
98	MMP2	THBS2
99	MMP2	TIMP2
100	MMP2	TIMP3
101	MYH11	ACTA2
102	NEDD4	KIFC3
103	NEDD4	PLK2
104	NEDD4	PMEPA1
105	NEDD4	SULF1
106	PCOLCE	ANTXR1
107	PDGFRB	NEDD4
108	PDGFRB	PDGFD
109	PDGFRB	PDGFRA
110	PDLIM3	PALLD
111	PLAU	SERPINE1
112	SDC1	MMP14
113	SERPINE1	LRP1
114	SPARC	COL5A1
115	SPARC	CTSK
116	TGFB3	CTGF
117	THBS1	CD36
118	THBS1	COL5A1
119	THBS1	LRP1
120	THBS1	MMP2
121	THBS1	PCOLCE
122	THBS1	SPARC
123	THBS1	TNFAIP6
124	TIMP2	MMP14
125	TIMP3	ADAMTS5
126	TIMP3	EFEMP1

127	TIMP3	MMP14
128	VCAM1	FLNC
129	VCAN	CXCL12
130	VCAN	FBLN2
131	VCAN	FBN1
132	WISP1	DCN

Supplementary Table 6. Over-represented EMT linked Gene Ontology biological process terms in the *DNM3OS* associated protein-interaction networks

**Biological process**	**Number of genes**	**Nominal *P***	**Adjusted *P*** [$]
Extracellular matrix organization	24	9.88E-25	7.33E-22
Cell substrate adhesion	16	1.22e-13	7.67E-12
Cell migration	27	1.33E-13	7.98E-12
Cell morphogenesis involved in differentiation	23	9.29E-12	3.96E-10

[$] *P*-values were adjusted using the Benjamini-Hochberg (1995) multiple testing correction method.

Supplementary Table 7. Over-represented EMT linked pathways in the *DNM3OS* associated protein-interaction networks

**KEGG pathways**	**Number of genes**	**Nominal *P***	**Adjusted *P*** [$]
ECM receptor interaction	18	6.35E-32	2.22E-30
Focal adhesion	20	1.87E-28	3.27E-27
TGFβ signaling pathway	8	8.33E-12	4.86E-11
Regulation of actin cytoskeleton	6	5.23E-06	2.29E-05
Gap junction	3	0.0008	0.0016
MAPK signaling pathway	4	0.0023	0.0038
Cell adhesion molecules (CAMs)	3	0.0026	0.0041
Calcium signaling pathway	3	0.0058	0.0079
P53 signaling pathway	2	0.0086	0.0108

[$] *P*-values were adjusted using the Benjamini-Hochberg (1995) multiple testing correction method.
