# Supplementary material for: Decoding critical long non-coding RNA in ovarian cancer epithelial-to-mesenchymal transition
Source: Nat Commun. 2017 Nov 17;8:1604. doi: 10.1038/s41467-017-01781-0 (PMC5693921; doi:10.1038/s41467-017-01781-0)
Supplement: Supplementary file 3 — Description of Additional Supplementary Files [file 41467_2017_1781_MOESM3_ESM.pdf]

Description of Additional Supplementary Files

File Name: Supplementary Data 1
Description: Ovarian cancer subtype information of TCGA patient cohort.

File Name: Supplementary Data 2
Description: Ovarian cancer subtype information of GSE9891 patient cohort.
